# The Relationship between Health-Promoting Behaviors and Resilience in Patients with Chronic Kidney Disease

**DOI:** 10.1155/2013/124973

**Published:** 2013-03-25

**Authors:** Li-Ching Ma, Hong-Jer Chang, Yueh-Min Liu, Hsiang-Li Hsieh, Lan Lo, Mei-Yu Lin, Kuo-Cheng Lu

**Affiliations:** ^1^Department of Nursing, Cardinal Tien Hospital, New Taipei, Taiwan; ^2^Graduate Institute of Long-Term Care, National Taipei College of Nursing, Taipei, Taiwan; ^3^Division of Nephrology, Department of Medicine, Cardinal Tien Hospital, School of Medicine, Fu-Jen Catholic University, 362 Chung-Cheng Road, Hsin-Tien, New Taipei, Taiwan

## Abstract

This cross-sectional research study explored differences in health-promoting behavior and resilience among three groups of chronic kidney disease patients (high-risk, early chronic kidney disease; early CKD and pre-end stage renal disease; pre-ESRD) treated at the Nephrology outpatient clinic in northern Taiwan. A total of 150 CKD outpatients were interviewed using structured questionnaires including a CKD Health to Promote Lifestyle Scale, and resilience scale. We found that the pre-ESRD group had lower resilience than either high-risk or early CKD groups. Factors affecting pre-ESRD resilience were gender, occupational status, diabetes and health-promoting behaviors. Factors affecting resilience of the high-risk group included level of education and health-promoting behaviors while factors affecting resilience in the early CKD group involved whether they are employed and health promoting behaviors. A significant positive correlation was found between health promoting behavior and resilience in all study subjects. Multiple regression analysis found that factors which could effectively predict resilience in patients at high-risk for CKD were gender, whether the patient had a job, nutrition, self-actualization, and stress level, accounting for 69.7% of the variance. Therefore, nursing education should focus on health promotion advocacy throughout the life of not only patients but also their families.

## 1. Introduction

According to the 2009 National Institutes of Health Survey, performed on a prospective cohort of 462,293 subjects in Taiwan, approximately 12% of subjects surveyed suffered from some form of chronic kidney disease (CKD). This survey also found that high blood pressure, diabetes, age over 65 years, a family history of disease, failure to give sufficient attention to health promotion, and other risk factors were responsible for the rise in the number of CKD cases annually [1, 2]. Once patients are on dialysis, they are exposed to the physical, psychological and social discomforts of the treatment [3]. In addition, there are secondary physical, psychological, and social burdens on their family members [4].

Resilience of individuals can mitigate the pressure caused by the negative impact of chronic disease. Intelligence, interpersonal skills, self-efficiency, positive response to the problem, and social support networks can all improve the patient's response to illness [5]. CKD patients and their families need to understand the importance of resilience, and medical personnel need to be educated in this area as well, in order to provide timely correct messaging that will help reduce the stress and frustration associated with chronic disease. An important factor affecting resilience in patients with CKD is the failure to give sufficient attention to health-promoting behaviors such as good nutrition, self-realization, stress reduction, proper sports, and fitting leisure time. Ignorance of such health promoting behaviors can lead to increased morbidity and mortality and even suicidal behavior [6, 7].

When CKD progresses to end stage renal disease (ESRD), patients must receive dialysis treatment to survive, and they are often prone to emotions such as a feeling of helplessness, depression, and fear. In a study by Yeh et al. [8], 12.3% of patients with CKD were in a depressed state as their family members assumed the role of primary caregiver, primary caregiver which altered their domesticity. According to a study by Liu et al. [9], more attention should be paid to the needs of families to assist in the prevention or treatment of disease. Since 2003, the Bureau of National Health Insurance (BNHI) in Taiwan, in response to the growing number of patients with ESRD and the subsequent heavy financial burden placed on BNHI, has implemented several pre-ESRD preventive plans and patient education programs (including high-risk health management) to actively promote the health of high-risk and kidney transplant patients. In addition, they sought to improve the health of family members of such high-risk groups, including spouses, parents, children, and siblings. Early detection and active treatment may effectively reduce the incidence of ESRD and enhance health management capabilities of such high-risk groups, thereby possibly increasing resilience.

Therefore, we strive to understand the effect of resilience on the management of such high-risk patients and their families in order to provide medical personnel with sufficient information to improve the welfare of CKD patients.

## 2. Methods

### 2.1. Study Design and Population

The study was approved by the institutional review board (CTH-100-3-5-007). Informed written consent was obtained from all subjects or their guardians after permission was obtained.

This study compared the differences in health-promoting behavior and resilience among three groups of CKD patients (high risk, early CKD, and pre-ESRD) treated at the nephrology outpatient clinic (OPD) in northern Taiwan. A cross-sectional descriptive design was used. CKD patients and their families (as a high-risk group) were recruited from April 1 to December 31, 2011, and divided into three groups by using the following inclusion criteria: (1) the high-risk group of CKD; (2) early CKD group stages 1, 2, and 3a: eGFR > 45 mL/min/1.73 m^2^ (by MDRD 4 variables equation); (3) pre-ESRD group involving CKD stages 3b, 4, and 5: eGFR < 45 mL/min/1.73 m^2^. Patients were also required to speak either Mandarin, Taiwanese, or Hakka language and had to be willing to sign a consent form.

### 2.2. Data Collection Procedures

After the researchers explained the study to the patients, 160 CKD patients who were recruited from the Nephrology OPD from Cardinal-Tien Hospital in New Taipei city, Taiwan, were pretested in face-to-face interviews. These interviews lasted approximately 30 to 60 minutes, including the time to fill out the resilience scale, record demographic characteristics and laboratory data from the medical records, and distribute additional questionnaires. Ten patients were excluded due to other illness with resultant 150 patients enrolled in the study. 

To estimate the number of samples, according to the G power 3.0 software, alpha was set at .05, the statistical power was set at .80, effect (effect size) was set at .25 (small to medium intensity), and the estimated number of samples was estimated at 120. Since a total of 150 patients were actually enrolled, this increased the power to .92.

### 2.3. GFR Calculation

This study used a structured questionnaire as a research tool which contained demographic characteristics, disease characteristics, health promotion lifestyle scale, and a resilience scale. 

(1) The demographic characteristics included gender, age, educational level, and occupation. 

(2) The disease characteristics included serum creatinine level, glomerular filtration rate (GFR), the level of CKD, and history of either high blood pressure or diabetes. 

The GFR formula, adapted from the simplified MDRD (Modification of Diet in Renal disease) formula, was used as follows:

[eGFR mL/min/1.73 m^2^ (Simplified MDRD) = 186 × Scr − 1.154 × Age − 0.203 × 0.742 (if female) × 1.212 (if black patient)].

(3) Health promotion lifestyle scale. Health promoting lifestyle was measured using the CKD Health to Promote Lifestyle Scale, which was considered valid and reliable. The validity and reliability of the original Health Promoting Lifestyle Scale were assessed using a sample of 920 subjects with a resulting Cronbach's *α* of .92 [10]. The CKD Health Promoting Lifestyle Scale comprised 40 items that assessed the following six subsets of lifestyle: (1) nutritional (5 questions); (2) health responsibility (8 questions); (3) self-actualization (8 questions); (4) interpersonal support (6 questions); (5) sports and leisure (6 questions); (6) stress management (7 questions). 

To validate the content, two nephrologists, a nurse specialist, a hemodialysis center head nurse, and a CKD health education management expert rated the questionnaires based on topic clarity and usability ratings, with a resulting content validity index value (CVI) of .90, consistent with a Cronbach's *α* of .93, with subsets between .79 and −.90. The measuring instrument used to obtain the frequency of reported lifestyle was a self-reporting Likert scale with a four-point response format of “never, sometimes, usually, and always,” with a rating score ranging from 0 to 3, and a total score of 0 to 120 (with higher scores indicating improved health promotion lifestyle).

(4) Resilience scale. Wagnild and Young [11] developed the resilience scale questionnaire used in this study. This scale was translated into Chinese by Li [12] and the Chinese version of the questionnaire was used in this study. The scale consisted of 25 questions covering the following five levels: (1) meaning of life (7 questions); (2) calm mind areas (6 questions); (3) retains confidence areas (6 questions); (4) indomitable spirit of the scope (3 questions); (5) acceptance of the existence of solitude areas (3 questions). One point was used by patients to “strongly disagree” and up to seven points to “strongly agree” in order to reflect the level most in line with the current situation, for a total score of 25–175 points (with higher scores indicating better resilience). Li [12] tested 329 college students with a resulting Cronbach's *α* value of .90; Wagnild and Young [11] used the scale as a test object for older women and achieved a Cronbach's *α* value of .94. Margaret [13] tested 277 elderly subjects with a resulting Cronbach's *α* value of .94. In this study, we achieved a Cronbach's *α* value of .89, which indicated that this scale had good reliability and validity for assessing patients' resilience.

### 2.4. Statistical Analysis

Statistical analysis included descriptive statistics (frequency, percentage, mean, and standard deviation) and inferential statistics that use independent samples *t*-test and one way analysis of variance (ANOVA) to determine the differences in participant perceptions of health promotion behavior, and resilience. Correlation and multiple regression analysis between demographic data, disease characteristics, health-promoting behavior subgroup (subset) and resilience were explored further. A *P* value less than 0.05 was considered statistically significant. All statistical analysis was performed using SPSS software (SPSS Version 19).

## 3. Results

### 3.1. Comparative Demographics, Disease Characteristics and Resilience of the Study Sample

Out of a total of 150 participants, 40 were high-risk group subjects. The average age of the high-risk group was 59.9 ± 14.6 years and their average GFR was 83.3 mL/min/1.73 m^2^. The average age of the 50 participants in the early CKD group was 65.1 ± 13.6 years and their average GFR was 75.6 mL/min/1.73 m^2^. The pre-ESRD group included 60 participants with an average age of 68.9 ± 14.6 years and an average GFR of 21.8 mL/min/1.73 m^2^. Among the 150 subjects, most were married, unemployed, and had diabetes.

#### 3.1.1. Difference in Resilience within Each Study Group (Table 1)

In high-risk groups, patients with higher education level (above high school) have a higher resilience score when compared with patients with lower education levels (*P* < .05). In both early CKD and pre-ESRD groups, employed patients have a higher resilience score than un employed patients (*P* < .05 versus *P* < .001 resp.). In pre-ESRD group, patients with diabetes have a lower resilience score when compared with patients without diabetes (*P* < .01).

#### 3.1.2. Difference in Resilience among the Three Study Groups

The resilience among the three groups was influenced by demographic characteristics. Chi-square analysis of the three groups found that various educational levels (*χ* ^2^ = 7.714, *P* < .05), with or without an occupation (*χ* ^2^ = 6.818, *P* < .05) and the presence or absence of diabetes (*χ* ^2^ = 16.617, *P* < .001), were significantly influenced on resilience score. 

### 3.2. Comparative Resilience of the Study Subjects

 For all subjects, the average resilience was 139.0 ± 21.1. Post hoc comparisons (Scheffe test) showed that the early CKD group's resilience score was higher than the other two groups' scores. The lowest score was found in the pre-ESRD patients (130.7 ± 22.1) (Table 2).

### 3.3. Comparison of Health Promotion Behavior of the Study Subjects

The overall health-promoting behaviors among different groups were significantly different (*F* = 6.982, *P* < .01). The health promotion behavior score of the pre-ESRD group was significantly lower than the other two groups. The sequencing of average score (from high to low) was nutrition, self-actualization, interpersonal support, stress management, health responsibility, and sports and leisure levels. The sequencing of average score (from high to low) of the early CKD group was nutrition, interpersonal support, self-actualization, stress management, health responsibility, and sports and leisure level. The sequencing of average score (from high to low) of the high-risk group was self-realization, stress management, nutrition, health responsibility, interpersonal support, and sports and leisure levels. Among the three groups, nutrition, self-actualization, interpersonal support, sports and leisure, and stress management levels were significantly different (*P* < .01) (Tables 3 and 4).

### 3.4. Correlation between Demographic Characteristics, Disease Characteristics, Health-Promoting Behavior, and Resilience

Table 5 shows that in the high-risk group, the resilience strongly correlated with education level, nutrition, self-realization, and stress management. In the early CKD group, the resilience correlated with, whether or not the subject was employed, nutrition, self-realization, interpersonal support, sports and leisure, and stress management levels. In the pre-ESRD group, the resilience correlated with gender, whether or not the subject was employed, diabetes, nutrition, self-realization, interpersonal support, sports and leisure, and stress management levels.

### 3.5. Evaluating the Predictive Variables of the Resilience

All the resilience variables were entered into the regression equation to advance into a set of virtual variables for each reference group. The results of the multiple regression analysis (*F* = 28.817, *P* < .001) found that gender, presence or absence of an occupation, nutrition level, self-actualization level, and stress management level were the variables that could meaningfully predict resilience and were found to explain 69.7% of the total variance. From standardized regression coefficient *β* value, stress management level had the strongest explanatory power (*β* = .349). The second strongest was self-actualization level. Under the control of other variables, stress management level (*B* = 1.794, *t* = 4.582, *P* < .001) significantly influences the resilience with each one unit increase in stress management accompanied by an increase in 1.794 units of resilience. Other variables such as gender, presence or absence of an occupation, nutrition level, and self-actualization level variables also reached statistically significance (Table 6).

## 4. Discussion

We found that resilience in the pre-ESRD group was lower than the other two groups. In the pre-ESRD patient group, the presence of diabetes also was higher than the other two groups. These patients required long-term dependence on drugs and diet to control the disease, which limited activities and may have increased psychological burden, causing the resilience scores to drop. Similarly, the study by Margaret [13] found that psychological dysfunction reduced resilience. Therefore, health problems related to the pre-ESRD group of patients require more attention from health care providers. 

 When health-promoting behaviors were compared, the pre-ESRD group scored lower than the other two groups. This may be due to the older age and more diabetes in the pre-ESRD group. In the nutrition subset of health promotion behavior, the early CKD group and pre-ESRD group performed better than high-risk group. Regarding the nutrition levels, the early CKD group and pre-ESRD group were provided appropriate amounts of protein, and low phosphorus, low potassium, but limited amounts of water and pickled foods. The high rate of implementation of this type of nutrition may be secondary to the establishment of CKD health education clinics in our hospital since 2005. Patients with kidney disease were provided health knowledge by a physician, nutritionist, and educator, who gave nursing advice and diet education along with regular monitoring of blood biochemistry values. Previous studies also demonstrated the importance of good nutrition and healthy lifestyle in maintaining renal function in CKD patients [14]. Powe et al. [15] also recommended that patients should implement a health promoting lifestyle to avoid diseases and their complications. 

Regarding self-actualization levels, the high-risk group scored better than the other two groups. In the study by Liu et al. [9], the families who cared for hemodialysis patients for more than three years understood the unique demands of the disease and accepted the impact of the disease on their own lives, resulting in better self-actualization levels. 

The level of sports and leisure was the worst health-promoting behaviors which are a common phenomenon present in all countries [16]. In all our study groups, sports and leisure levels subset of health-promoting behaviors was below average. The mean age of CKD and pre-ESRD subjects were older than 65 years in our study, affecting the physical strength to engage in sports and leisure activities. Therefore, the sports and leisure scores were lower compared to the high-risk group of patients.

A study by Mary et al. [17] showed an increase in resilience after a four-week resilience course. Thus, the determinant factors of resilience need to be explored. Our results showed that the three groups shared common predictable resilience factors with respect to gender and occupational status. The nutrition, self-actualization, and stress management subsets of health promotion behaviors were found to predict resilience. Regarding gender, the results of this study found that the resilience in males was higher than in females, similar to the findings of Li [12], in contrast to Grotberg's report [18] that the divorce can also affect resilience. Faced with the predicament of divorce, the recovery abilities of the female respondents were greater as they were more likely to seek help, share feelings, and indulge in other interactive features, compared to the male respondents, who focused more on the causes of the problem and how to resolve them. 

Occupational status was another factor affecting the resilience of CKD patients. We found that patients with an occupation had better resilience, similar to the results of Ross and Wu [19]. CKD patients and high-risk groups also have special nutritional needs which are particularly important, as shown by Yang [14], for both the prevention and treatment of CKD. Another study by Hollingdale et al. [20] demonstrated that 80% of CKD patients were willing to accept restrictions on protein in order to reduce the hazard of ESRD. Self-actualization and stress management are psychological protective factors and best related to resilience [9, 21]. In interviews with CKD patients, Liu et al. [9] and Lamond et al. [21] found that CKD patients admitted that they could not enjoy food like a normal person, and, unfortunately, a degree of freedom was no longer theirs. Even so, they were willing to abide by the dietary restrictions in order to avoid the entrance of dialysis treatment. Lee [22] and Liu [23] have shown that in patients with CKD, the disease itself can cause a great deal of stress. These chronic stresses can lead to depression symptoms. Thus, the future can be enhanced in patients with CKD when the cognitive aspects of adjustment are discussed and hobbies are pursued to reduce stress and allow patients to reconsider their own roles and participate in a group health education to strengthen the ability to adapt to chronic stress. In this way, health education may contribute to the resilience of CKD patients.

 The study was limited by time and several research factors. Since it was only a cross-sectional study, there was no long-term followup or continuity to assess patients for resilience at each stage of the kidney disease. Also, we enrolled only those high-risk and CKD patients who utilized the outpatient facilities at our hospital. Therefore, our findings regarding health promotion and resilience cannot be generalized to other hospital outpatient settings.

## 5. Conclusion

 In the early CKD group, the factors affecting resilience included occupation and health-promoting behaviors. However, factors affecting resilience in the pre-ESRD group included gender, occupation, diabetes, and health-promoting behaviors. In the CKD patients, resilience was a significant positive correlation with health promotion behavior. Factors which could effectively predict resilience were gender, occupation, nutrition, self-actualization, and stress levels. Therefore, health education of clinical nurses should focus on health promotion advocacy throughout the life of not only patients, but also their families. We hope that the results of this study can be used as a reference to promote healthy behavior and improve resilience among CKD patients.

## Figures and Tables

**Table 1:** Difference in resilience between population characteristics and disease characteristics among study groups (*N* = 150).

Variables	High-risk group *N* = 40				Early CKD group *N* = 50				Pre-ESRD group *N* = 60				*χ* ^2^
	*n*	(%)	Mean ± SD	*t* values	*n*	(%)	Mean ± SD	*t* values	*n*	(%)	Mean ± SD	*t* values
Gender				.461				1.847				2.756**	*χ* ^2^ = 5.193
Male	16	40.0	143.56 ± 13.18		32	64	150.56 ± 17.50		33	55	137.67 ± 19.07		
Female	24	60.0	141.88 ± 9.98		18	36	138.39 ± 29.23		27	45	122.37 ± 23.12		
Education level				−2.276*				−1.852				−1.258	*χ* ^2^ = 7.714*
≤junior school	16	40.0	137.81 ± 10.90		24	48	140.08 ± 19.18		40	66.7	128.25 ± 19.86		
≥high school	24	60.0	145.71 ± 10.51		26	52	151.81 ± 24.95		20	33.3	135.85 ± 26.01		
Marital status				−1.225				.682				−1.047	*χ* ^2^ = .506
Single	7	17.5	137.86 ± 9.94		11	22	150.36 ± 17.41		14	23.3	125.36 ± 28.16		
Married	33	82.5	143.55 ± 11.38		39	78	145 ± 24.31		46	76.7	132.43 ± 20.09		
Occupation				.09				2.422*				4.479***	*χ* ^2^ = 6.818*
Yes	8	20.0	142.88 ± 9.49		20	40	154.30 ± 12.15		12	20	147.33 ± 11.46		
No	32	80.0	142.47 ± 11.76		30	60	140.77 ± 16.74		48	80	126.65 ± 22.34		
Diabetes				−1.469				.149				−2.931**	*χ* ^2^ = 16.617***
Yes	10	25.0	137.78 ± 9.92		9	18	147.22 ± 18.19		31	51.7	123.19 ± 22.91		
No	30	75.0	143.94 ± 11.38		41	82	145.95 ± 24.02		29	48.3	138.90 ± 18.48		
Hypertension				.816				−.130				1.689	*χ* ^2^ = 5.821
Yes	3	7.5	146.4 ± 12.18		17	34	145.59 ± 21.45		18	30	138.06 ± 20.47		
No	37	92.5	142 ± 11.17		33	66	146.48 ± 23.96		42	70	127.67 ± 22.37		

High-risk group: the families of CKD patients.

Early CKD group: GFR > 45 mL/min/1.73 m^2^.

Pre-ESRD group: GFR < 45 mL/min/1.73 m^2^.

*P < .05, **P < .01, and ***P < .001.

Statistical methods: descriptive statistics, *t* tests (within group), and *χ* ^2^ tests (between groups).

**Table 2:** Difference in resilience among study groups (*N* = 150).

Variables	*N*	Mean	Standard deviation	Minimum	Maximum	*F* value
All	150	139.05	21.171	49	171	8.788***
High-risk group	40	142.55	11.234	110	164	Early CKD group >
Early CKD group	50	146.18	22.918	50	171	High-risk group >
Pre-ESRD group	60	130.78	22.170	49	169	Pre-ESRD group

High-risk group: the families of CKD patients.

Early CKD group: GFR > 45 mL/min/1.73 m^2^.

Pre-ESRD group: GFR < 45 mL/min/1.73 m^2^.

*P < .05, **P < .01, and ***P < .001.

Statistical methods: descriptive statistics, one way ANOVA.

**Table 3:** Difference in subgroups of health promotion behavior among study groups (*N* = 150).

	All	High-risk group	Early CKD group	Pre-ESRD group	
Variables	*N* = 150	*N* = 40	*N* = 50	*N* = 60	*F* value
	Mean ± Standard deviation	Mean ± Standard deviation	Mean ± Standard deviation	Mean ± Standard deviation	
Nutrition	10.94 ± 2.742	12.27 ± 2.375	11.24 ± 2.544	9.8 ± 2.698	11.691***
Health responsibility	13.47 ± 4.587	13.65 ± 4.638	14.12 ± 4.623	12.8 ± 4.509	1.175
Self-actualization	17.49 ± 4.587	19.38 ± 4.210	18.24 ± 5.255	15.6 ± 4.951	8.097***
Interpersonal support	12.51 ± 3.874	13.27 ± 3.449	13.18 ± 3.921	11.43 ± 3.684	4.023**
Sports and leisure	5.34 ± 3.941	7.35 ± 4.073	4.8 ± 3.870	4.45 ± 3.466	7.868**
Stress management	14.15 ± 4.119	15.4 ± 2.734	14.64 ± 4.663	12.92 ± 4.126	5.159**
Total (health promotion behavior)	73.83 ± 18.909	80.47 ± 15.733	76.32 ± 19.845	67.32 ± 18.275	6.982**

High-risk group: the families of CKD patients.

Early CKD group: GFR > 45 mL/min/1.73 m^2^.

Pre-ESRD group: GFR < 45 mL/min/1.73 m^2^.

*P < .05, **P < .01, and ***P < .001.

Statistical methods: descriptive statistics, one way ANOVA.

**Table 4:** Average scores and order of subets of health promotion behavior (*N* = 150).

Variables	Number of questions	High-risk group		Early CKD group		Pre-ESRD group	
		*N* = 40		*N* = 50		*N* = 60	
		Average	Sequencing	Average	Sequencing	Average score	Sequencing
Nutrition	5	2.454	3	2.248	1	1.960	1
Health responsibility	8	1.706	4	1.765	5	1.600	5
Self-actualization	8	3.230	1	2.280	3	1.950	2
Interpersonal support	6	1.659	5	2.197	2	1.905	3
Sports/leisure	6	1.050	6	0.800	6	0.742	6
Stress management	7	2.570	2	2.091	4	1.846	4

High-risk group: the families of CKD patients.

Early CKD group: GFR > 45 mL/min/1.73 m^2^.

Pre-ESRD group: GFR < 45 mL/min/1.73 m^2^.

*P < .05, **P < .01, and ***P < .001.

Statistical methods: descriptive statistics.

**Table 5:** Correlation between resilience and demographic characteristics, disease characteristics, and subgroups of health-promoting behavior (*N* = 150).

	Resilience scores		
Variables	High-risk group	Early CKD group	Pre-ESRD group
	*N* = 40	*N* = 50	*N* = 60
	*r*	*r*	*R*
Gender	−.075	−.239	−.345**
Education level	.349*	.244	.163
Occupation	−.015	−.282*	−.377**
Diabetes	.232	−.015	.358**
Nutrition	.406**	.665**	.443**
Self-actualization	.551**	.820**	.690**
Interpersonal support	.257	.736**	.533**
Sports/leisure	.193	.443**	.419**
Stress management	.379*	.769**	.680**

High-risk group: the families of CKD patients.

Early CKD group: GFR > 45 mL/min/1.73 m^2^.

Pre-ESRD group: GFR < 45 mL/min/1.73 m^2^.

*P < .05, **P < .01, and ***P < .001.

Statistical methods: correlation analysis.

**Table 6:** Regression analysis of resilience (*N* = 150).

Predictive variables	Unstandardized coefficient		Standardized coefficients	*t* values	*P*	*R* ^2^	Adjusted *R* ^2^
	The estimated value of the *B*	Standard error	*β* distribution				
Gender	−5.892	2.178	−.139	−2.705	.008		
Occupation	−7.152	2.493	−.150	−2.869	.005		
Nutrition	1.227	.509	.159	2.410	.017		
Self-actualization	1.214	.361	.292	3.360	.001		
Stress management	1.794	.392	.349	4.582	.000		

Total						.697	.673

Statistical methods: multiple regression analysis.
